# Musculoskeletal modelling of the human cervical spine for the investigation of injury mechanisms during axial impacts

**DOI:** 10.1371/journal.pone.0216663

**Published:** 2019-05-09

**Authors:** Pavlos Silvestros, Ezio Preatoni, Harinderjit S. Gill, Sabina Gheduzzi, Bruno Agostinho Hernandez, Timothy P. Holsgrove, Dario Cazzola

**Affiliations:** 1 Department for Health, University of Bath, Bath, United Kingdom; 2 Centre for Orthopaedic Biomechanics, Department of Mechanical Engineering, University of Bath, Bath, United Kingdom; 3 College of Engineering, Mathematics and Physical Sciences, University of Exeter, Exeter, United Kingdom; Medical College of Wisconsin, UNITED STATES

## Abstract

Head collisions in sport can result in catastrophic injuries to the cervical spine. Musculoskeletal modelling can help analyse the relationship between motion, external forces and internal loads that lead to injury. However, impact specific musculoskeletal models are lacking as current viscoelastic values used to describe cervical spine joint dynamics have been obtained from unrepresentative quasi-static or static experiments. The aim of this study was to develop and validate a cervical spine musculoskeletal model for use in axial impacts. Cervical spine specimens (C2-C6) were tested under measured sub-catastrophic loads and the resulting 3D motion of the vertebrae was measured. Specimen specific musculoskeletal models were then created and used to estimate the axial and shear viscoelastic (stiffness and damping) properties of the joints through an optimisation algorithm that minimised tracking errors between measured and simulated kinematics. A five-fold cross validation and a Monte Carlo sensitivity analysis were conducted to assess the performance of the newly estimated parameters. The impact-specific parameters were integrated in a population specific musculoskeletal model and used to assess cervical spine loads measured from Rugby union impacts compared to available models. Results of the optimisation showed a larger increase of axial joint stiffness compared to axial damping and shear viscoelastic parameters for all models. The sensitivity analysis revealed that lower values of axial stiffness and shear damping reduced the models performance considerably compared to other degrees of freedom. The impact-specific parameters integrated in the population specific model estimated more appropriate joint displacements for axial head impacts compared to available models and are therefore more suited for injury mechanism analysis.

## Introduction

The worldwide reported incidence for traumatic cervical spine injuries is 15 to 39 cases per million [1, 2]. Spinal injuries associated with permanent neurological damage have devastating consequences on the quality of life of the individual and can result in individual lifetime costs rising to $3 million [3]. Neurological damage can reduce quality of life and lead to secondary factors such as discrimination, depression, and suicide [3] with wider societal costs of up to $9.7 billion [4]. In sporting activities, cervical spine injuries are more common during high energy contact sports such as American football and Rugby union, where the incidence rates of catastrophic cervical spine injuries range from 2 to 10 per 100,000 players per year for American football [5] and Rugby union [6] respectively. A better understanding of injury mechanisms is key to educate coaching and conditioning as well as to inform possible changes to the governing rules of contact sports. *In silico* approaches allow the estimation of measures such as internal joint loads and muscle forces that are extremely difficult and impractical to safely measure in sporting conditions. Also, they give the opportunity to explore ranges of theoretical scenarios [7] and thus understand how changes in impact conditions (e.g. external load, movement technique) and neuromusculoskeletal characteristics (e.g. muscle activation patterns) may affect injury factors. The reliability of such computational approaches is strongly dependent on the models used and their rigorous validation [8]. Although a lot of work has been produced to investigate the mechanisms of cervical spine injury in impact events, such as motor vehicle accidents and falls [9, 10], application of musculoskeletal models in sporting neck injury research is lacking.

Musculoskeletal models of the cervical spine have been created for the biomechanical analysis of functional neck movements [11–13] and impacts [9, 10, 14, 15]. Multibody musculoskeletal modelling can estimate system dynamics during sport impact events and, if rigorously validated, provide a viable approach to test fundamental principles and investigate their injury mechanisms [8]. This approach also enables a practical and direct use of experimental data as inputs for musculoskeletal analyses [16], and allows simulations to be run at low computational cost compared to detailed finite element models. Furthermore, musculoskeletal simulation results can be used as boundary conditions to finite element models [17], which can then provide a more detailed description of the stress and strain patterns experienced by specific spinal structures [18]. Musculoskeletal models of the cervical spine have incorporated biomechanical properties of the intervertebral disc to investigate and better understand head and neck injury mechanisms during dynamic loading scenarios such as motor vehicle collisions and falls [9, 10, 14, 15, 19, 20]. By approximating the complex dynamic behaviour of spinal joints the resulting joint motions can be estimated providing valuable information for injury mechanism analysis.

The viscoelastic behaviour of the intervertebral disc [21, 22] in musculoskeletal models has been represented with the Kelvin-Voight model of a parallel arrangement of a spring and a damper, which is referred to as a “bushing element” in the automotive sector [23, 24]. The stiffness and damping values of the bushings are obtained from *in vitro* experimental studies on human and animal (e.g. porcine, bovine) specimens which are implemented in musculoskeletal models [9, 10, 14, 15, 19, 25, 26]. Some of these musculoskeletal models have been used to analyse the internal loading of the cervical spine during axial compressive loading [10, 14], however the *in vitro* experimental procedures used to extract cervical joint stiffness values [27] were not conducted under conditions that correctly reflect the scenarios the models are used to evaluate [10, 14]. For example, the model initially developed by Camacho et al. [14] and later updated and used by Nightingale et al. [10] is used in the analyses of axial head impacts with peak forces of 2 kN being reached within 5 to 10 ms of loading. These values are an order of magnitude higher to what the study of Nightingale et al. [27] used, with peak loads of near 200 N reached in 2 s, to calculate joint stiffnesses and are not representative of high energy collisions occurring in sport. In fact, cervical spine injuries experienced during sport impacts are often caused by loads characterised by a high rate and magnitude of loading [7, 28].

From an experimental point of view, *in vitro* tests have often investigated the loading response of intervertebral discs using single motion segments (vertebra-disc-vertebra) under static or quasi-static loading conditions [29–32]. However, the behaviour of the entire cervical spine as a multi-segmented beam with interactions between joints is too complex to be modelled as the sum of individual joint responses to loading [27, 33]. The lack of a model that is representative of the cervical spine behaviour under impulsive axial loads is likely to be due to technical limitations in both experimental and computational approaches. The reliable estimation of individual joint stiffness of a multi-level cervical spine under such conditions would require experimental rigs capable of applying high load-controlled impulses whilst measuring individual vertebral motion. Currently experimental designs that can load a multi-level cervical spine specimen and measure individual joint displacements mechanically is challenging. However, combining subject specific modelling with high speed motion capture [33, 34] can be used to measure vertebral motion without the need for highly technical experimental rigs.

Therefore, the aim of this study was to: (a) estimate the viscoelastic properties of individual joints of multi-jointed cervical spines under loading conditions representative of sport impacts; (b) create and validate the first musculoskeletal model of the cervical spine that efficiently and reliably enables the estimation of compressive and shear joint forces and resulting motions via linear bushing (Kelvin-Voight) elements during impulsive loads; and (c) evaluate the newly developed model’s behaviour during an injurious sporting scenario.

## Materials and methods

*In vitro* experimental data and *in silico* methods were used to estimate the viscoelastic properties of the cervical spine’s joints. Representative loads of sub-catastrophic sporting impacts were applied to porcine cervical spine specimens (C2-C6) which were used as human specimen surrogates during experimental testing.

### *In vitro* experiments

Six porcine cervical spine specimens (C2-C6) were excised from pigs aged between 8 and 12 months at the time of slaughter (Larkhall Butchers, Bath, UK). Surrounding musculature was removed, facet capsules and ligaments were maintained apart from the anterior longitudinal ligament. Specimens were secured in a neutral position into nylon pots with bone cement (CMW, DePuy Int. Ltd., Leeds, UK). Motion capture markers (9 mm diameter) were glued using epoxy adhesive and allowed to become secure (approximately 10 minutes) in a non-collinear arrangement to the anterior surface of the vertebral bodies. Specimens were then wrapped in paper towels, sprayed with 0.9% saline solution, sealed in plastic bags and frozen at -24°C. Each frozen specimen underwent 0.1 mm resolution micro-computed tomography (μCT) scans (XT225 ST, Nikon Metrology, UK) prior to impact testing. The mass and height of each specimen were recorded and are presented in Table 1.

**Table 1:** Descriptive data of porcine cervical spine segments (C2-C6).

Specimen number	Mass (kg)	Height (m)
**S1**	0.378	0.203
**S2**	0.444	0.215
**S3**	0.396	0.202
**S4**	0.375	0.199
**S5**	0.358	0.202
S6*	0.570	0.223
**Mean ± SD**	0.390 ± 0.033	0.204 ± 0.006

* The mass and height of Specimen 6 (S6) are only shown for comparison and not included in the average values of the specimens as it sustained fractures at the C2, C3 and C4 vertebral levels.

**Fig 1:**
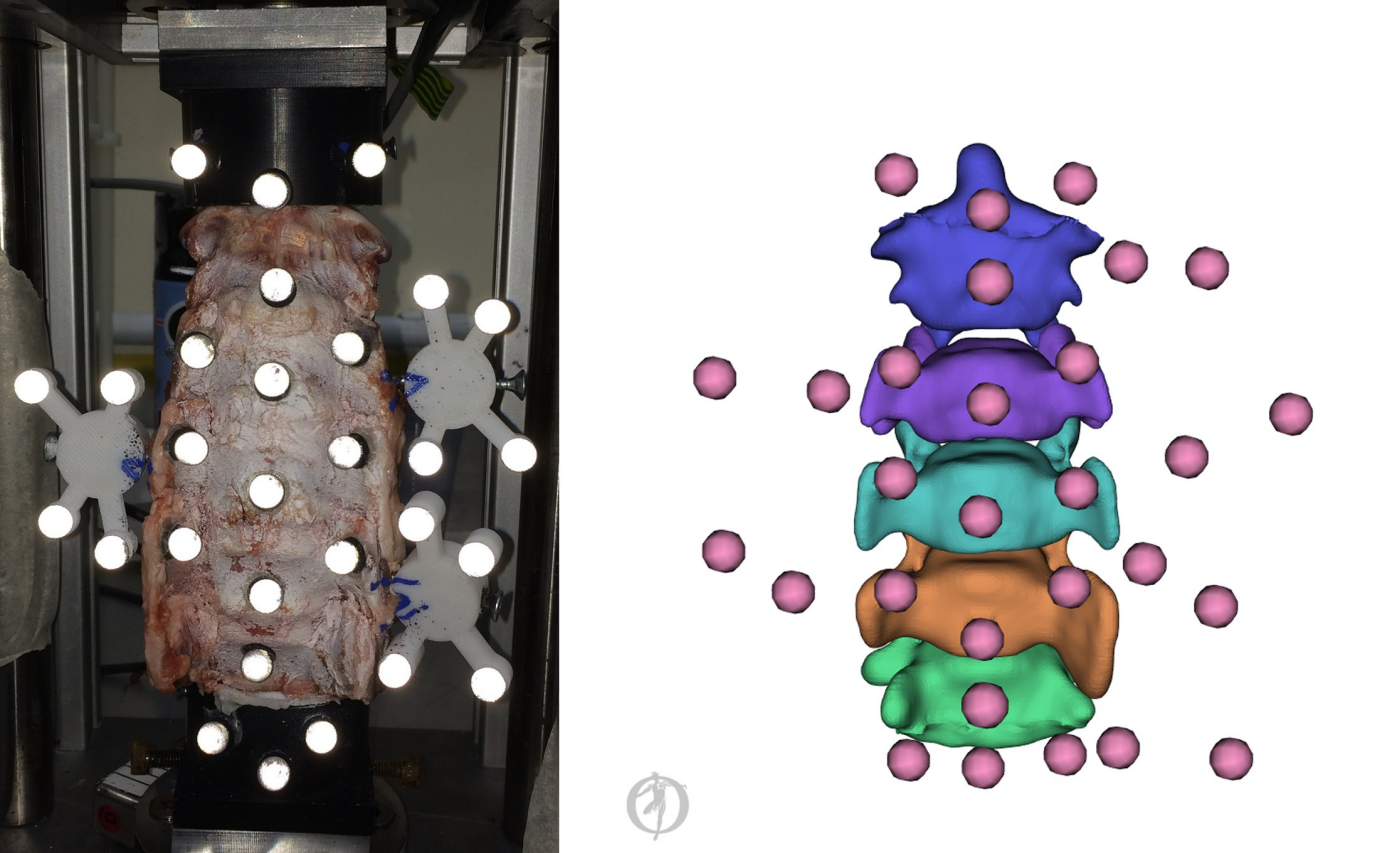
**Experimental set up of the spinal specimen positioned in the impact rig (left) and digital representation as a specimen specific model with virtual registered markers (right)**. Markers secured to the anterior aspect of the specimen and the cranial and caudal pots were used for the registration process during model creation. The markers of the cranial pot and the clusters secured to the C3, C4 and C5 vertebrae were used as tracking markers in the optimisation.

On the day of testing each specimen was left to thaw at room temperature (21 ± 2°C) whilst kept hydrated by applying saline solution to the surface of the wrapped specimens. The specimens did not undergo any preconditioning prior to impact testing. Motion capture tracking clusters were placed posteriorly to each transverse process of the C3, C4 and C5 vertebrae (Fig 1) and rigidly secured to the bony segments by means of a self-tapping screw. The specimen was mounted in a impactor [35] and was preloaded with 152 N via two constant force springs (51 N bilateral to the specimen) and the weight of the impact plate (50 N cranial to the specimen) [33, 35]. The experimental configuration constrained C2 to one DoF (axial translation) and C6 to zero DoF. This left the C3 to C5 vertebrae (C3-C4 and C4-C5 joints) unconstrained and able to move in a more physiologically manner.

A load of 80 N was dropped from a height of 0.5 m to the impact plate on the cranial aspect of the specimen to simulate peak forces measured during sub-catastrophic rugby tackles [36, 37]. Two 22 kN load cells (Model SLC41/005000, RDP Electronics Ltd., UK) were used to collect cranial and caudal force data at 1 MHz using an analogue to digital converter (TiePie Handyscope HS5 USB Oscilloscope, TiePie Engineering, Koperslagersstraat, Netherlands). Synchronised kinematic data were recorded by a five-camera motion capture system (Oqus, Qualisys, Sweden) at 4 kHz. Following impact testing specimens were μCT scanned to ensure the impact was sub-catastrophic.

Impact force data were filtered with a zero-lag fourth-order low-pass Butterworth filter with a cut-off frequency of 5 kHz (Matlab 2017a, The Mathworks, Natick, MA, USA). Kinematic marker data were filtered using the same filter with a cut-off frequency of 150 Hz after a power density analysis was performed on the raw kinematic data (Matlab 2017a). For both sets of data the time of impact was identified when the cranial load cell measurement exceeded 200 N [33, 35].

### Musculoskeletal model creation

The pre-impact μCT images were segmented (ScanIP M-2017.06, Simpleware, UK) to obtain specimen specific geometries of the cervical spine vertebrae. The MeshLab v2016.12 [38], NMSBuilder 2.0 [39] and OpenSim 3.3 [40] software packages were used to create specimen specific musculoskeletal models analogous to conventional methods used [41].

**Fig 2:**
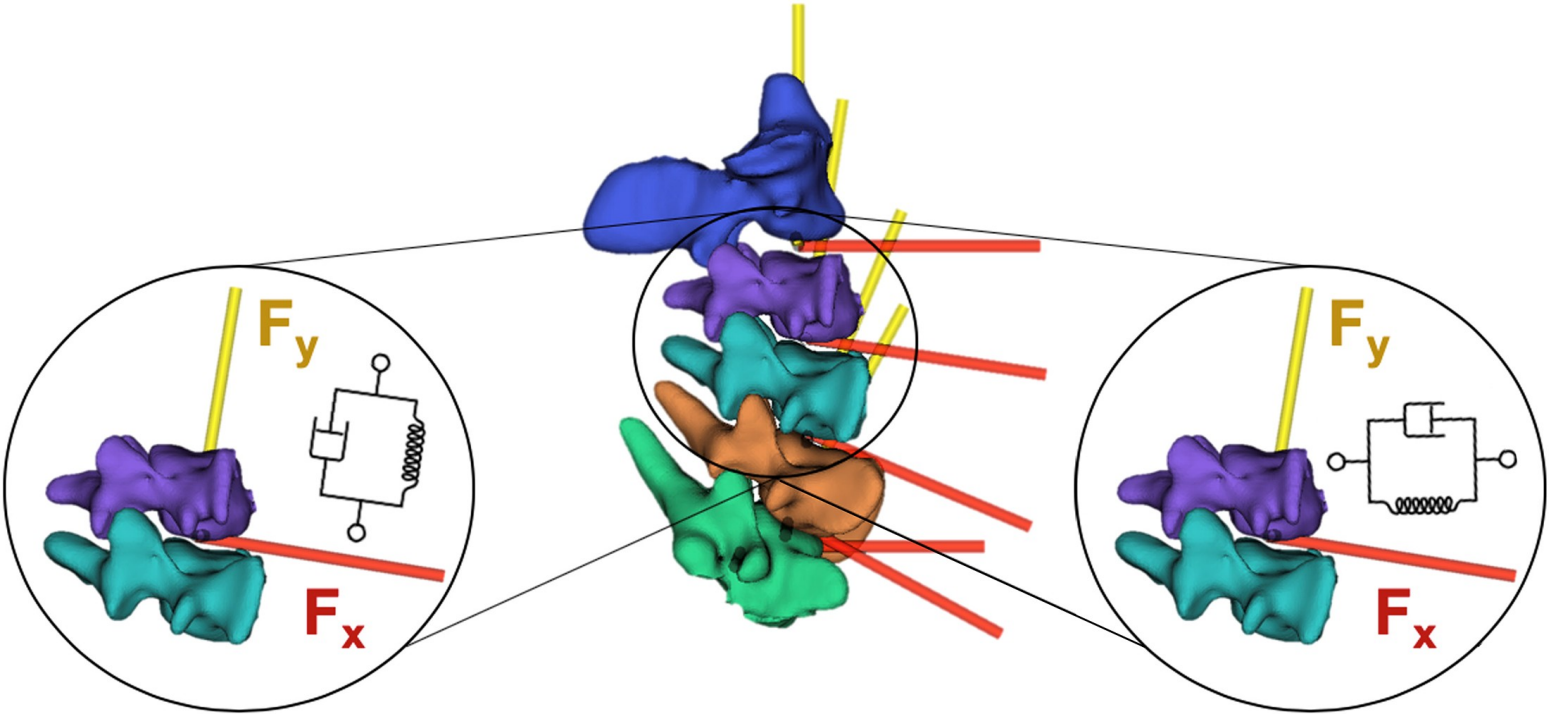
Joint and coincident 6 DOF viscoelastic bushing element locations. Only axial (Fy—left) and anteroposterior (Fx—right) viscoelastic elements were optimised, the parameters of the remaining four degrees of freedom remained at their initialised values.

Joint frame origins were located at the center of the intervertebral mid-planes between the inferior surface of the cranial segment and superior surface of the caudal segment for each of the four joints [42]. The anterior-posterior (x-axis) and medio-lateral (z-axis) axes were defined parallel to the superior surface of the caudal vertebrae with the superior-inferior (y-axis) axis normal to this plane (Fig 2). Six degree of freedom viscoelastic bushing elements comprised of a linear spring and damper in parallel (Kelvin-Voight model) were defined through the OpenSim 3.3 Matlab API to be coincident with the joint frames origins to overcome dynamic errors [43]. Reference values from the literature [9] were used to initialise all degrees of freedom of the four bushing elements. The OpenSim models were then constrained to replicate the experimental set up. Virtual markers were created in the same relative position as the experimental tracking markers to the cervical vertebrae by registering their position to the segmented static marker positions measured from the μCT scans.

### Optimisation pipeline

**Fig 3:**
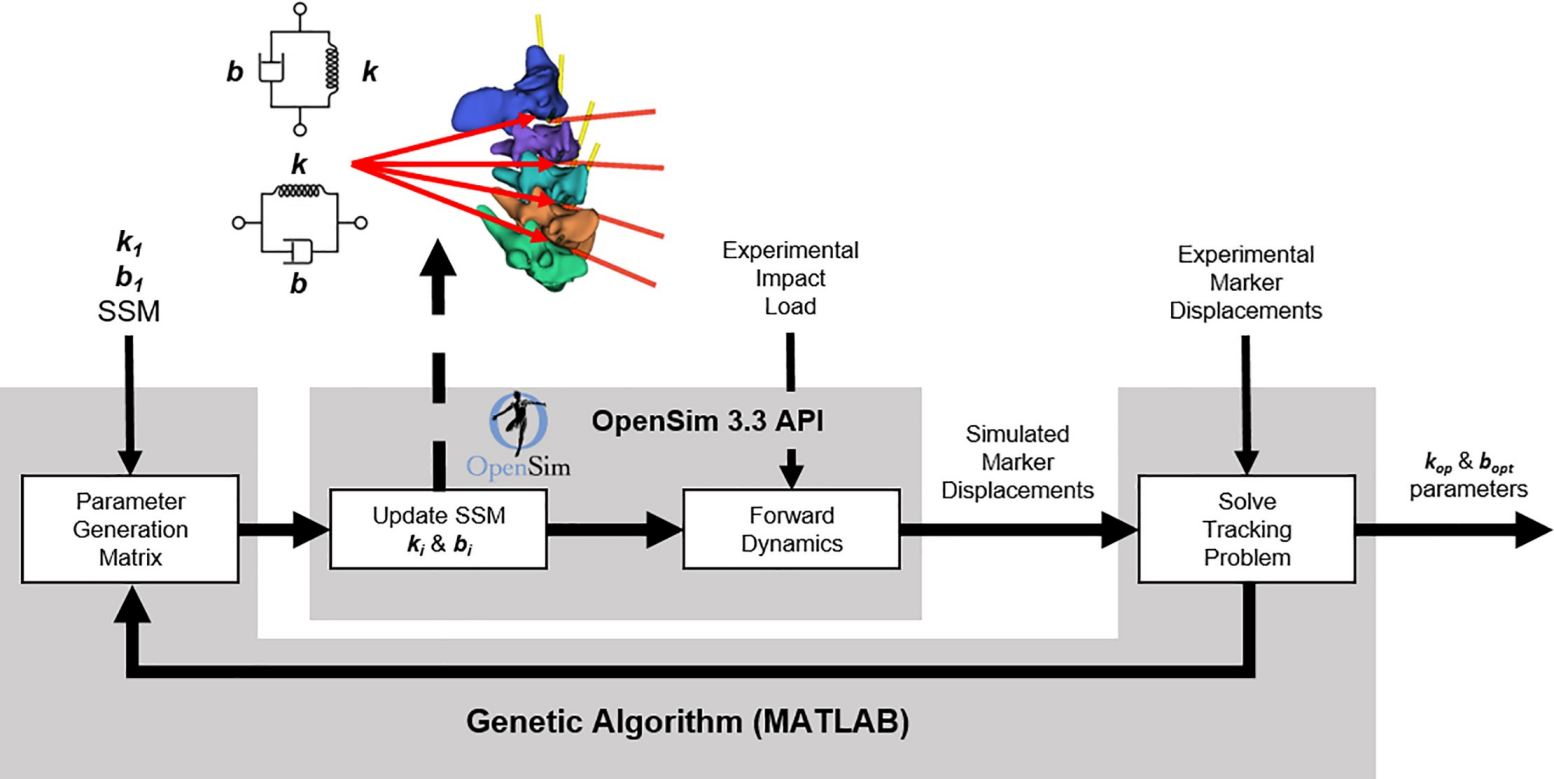
Optimisation pipeline used to estimate specimen specific model viscoelastic joint parameters. Literature values [9] (k_1_ and b_1_) were used to initialise the 6 DoF viscoelastic bushing elements of the specimen-specific models (SSM). A total of 16 optimised stiffness (k_opt_) and damping (b_opt_) for axial and shear degrees of freedom were estimated.

A dynamic optimisation pipeline (Fig 3) was developed to identify the optimal compressive (superior-inferior) and shear (anterior-posterior) viscoelastic bushing parameters. Simulations were performed up to 5 ms after the time of impact, which contained the cranial load peaks. A genetic algorithm (Matlab 2017a) was used to investigate the parameter space and identify the optimal viscoelastic bushing parameters (n = 16) by minimising the root mean square error (RMSE) between measured and simulated 3D marker kinematics over the 5 ms simulation window.

### Validation and sensitivity analysis

A five-fold cross validation was completed by applying the median value of the identified parameters obtained from four of the five spines to the model of the fifth spine iteratively a total of five times. The new combination of model and parameters was then used in the forward dynamic section of the previous pipeline (Fig 3) and evaluated against the experimental kinematic data of remaining fifth model, which was not included in the calculation of the parameters median value.

The 16 optimised parameters of each spine were grouped into four sets of four parameters dependent on their functionality: axial stiffness ($k_{y}=[k_{y}^{C2C3},k_{y}^{C3C4},k_{y}^{C4C5},k_{y}^{C5C6}]$), axial damping ($b_{y}=[b_{y}^{C2C3},b_{y}^{C3C4},b_{y}^{C4C5},b_{y}^{C5C6}]$), shear stiffness ($k_{x}=[k_{x}^{C2C3},k_{x}^{C3C4},k_{x}^{C4C5},k_{x}^{C5C6}]$) and shear damping ($b_{x}=[b_{x}^{C2C3},b_{x}^{C3C4},b_{x}^{C4C5},b_{x}^{C5C6}]$), where *k* is stiffness, *b* is damping, subscripts indicate direction (*y* compressive and *x* shear) and superscripts show the joint level. Model sensitivity to individual parameter set uncertainty was also assessed by varying individual sets from 50% to 150% of their identified optimum value.

To assess model sensitivity to combined changes in the four parameter sets a 1000 sample Monte Carlo analysis (Matlab 2017a) was performed by randomly perturbing axial stiffness, axial damping, shear stiffness and shear damping simultaneously with a uniform distribution between 50% and 150% of their identified optimum value.

$$p_{i}=p+rp$$

Where *p* is the entire set of optimised parameters *p* = [*k*_*y*_,*b*_*y*_,*k*_*x*_,*b*_*x*_], *r* = [−0.5,0.5] is the coefficient used to induce the parameter perturbations and *p*_*i*_ is the *i*^*th*^ set of perturbed parameters of the sensitivity analysis. Third degree polynomial surfaces were then fitted to the six pairs of parameter combinations to better asses their effect on RMSE change. Changes in the RMSE during the perturbations were evaluated as:

$$\Delta RMSE={RMSE}_{per}-{RMSE}_{opt}$$

Where *RMSE*_*per*_ and *RMSE*_*opt*_ are the RMSE between experimental and simulated marker kinematics of the *i*^*th*^ parameter set perturbation simulation of the Monte Carlo analysis and identified optimum parameter sets respectively.

Similarly, the joint frame position (JFP) on the intervertebral mid-planes was programmatically varied between -0.02 m and 0.02 m in the anteroposterior direction on all four joints simultaneously.

$${JFP}_{i}^{x}={JFP}^{x}+dx$$

Where *JFP*^*x*^ is the anteroposterior joint frame positions for the four cervical spine joints C2-C3, C3-C4, C4-C5, C5-C6, *d*_*x*_ = [−0.02,0.02] is the displacement in meters applied to the joint frame locations and ${JFP}_{i}^{x}$ is the newly defined anteroposterior position of the joint frames.

### Application to an injurious sporting scenario

The viscoelastic parameters estimated in this study, and previously used viscoelastic parameters from the literature [9] were then integrated in a population specific model (i.e. “Rugby Model”, [44]) to evaluate their behaviour during a sporting injurious scenario. This analysis was based on the comparison between three different models: i) the original “Rugby Model” [44] that utilises kinematic constraints [11], ii) a version implemented with the 6 DoF bushings from de Bruijn et al. [9] updated with the median values of the C3-C4 and C4-C5 joints for axial and shear viscoelastic parameters estimated in this study (hence referred to as impact-specific), and iii) the “Rugby Model” integrated with the original de Bruijn et al. [9] viscoelastic parameters (hence referred to as quasi-static).

The models response was compared during a simulated head-first impact in rugby, and consisted in the analysis of the cervical spine joint kinematics and reaction forces. Forward dynamic simulations (OpenSim 3.3) were used for the analysis and driven by a set of pure axial loads applied to the skull segment. Existing muscle actuators of the “Rugby Model” were included but no activation was prescribed to them. The external load profile used as input for the forward dynamics simulations was taken from dummy head forces (Hybrid III, Humanetics, Germany) measured during live scrum trials against an instrumented scrum machine [45].

## Results

**Fig 4:**
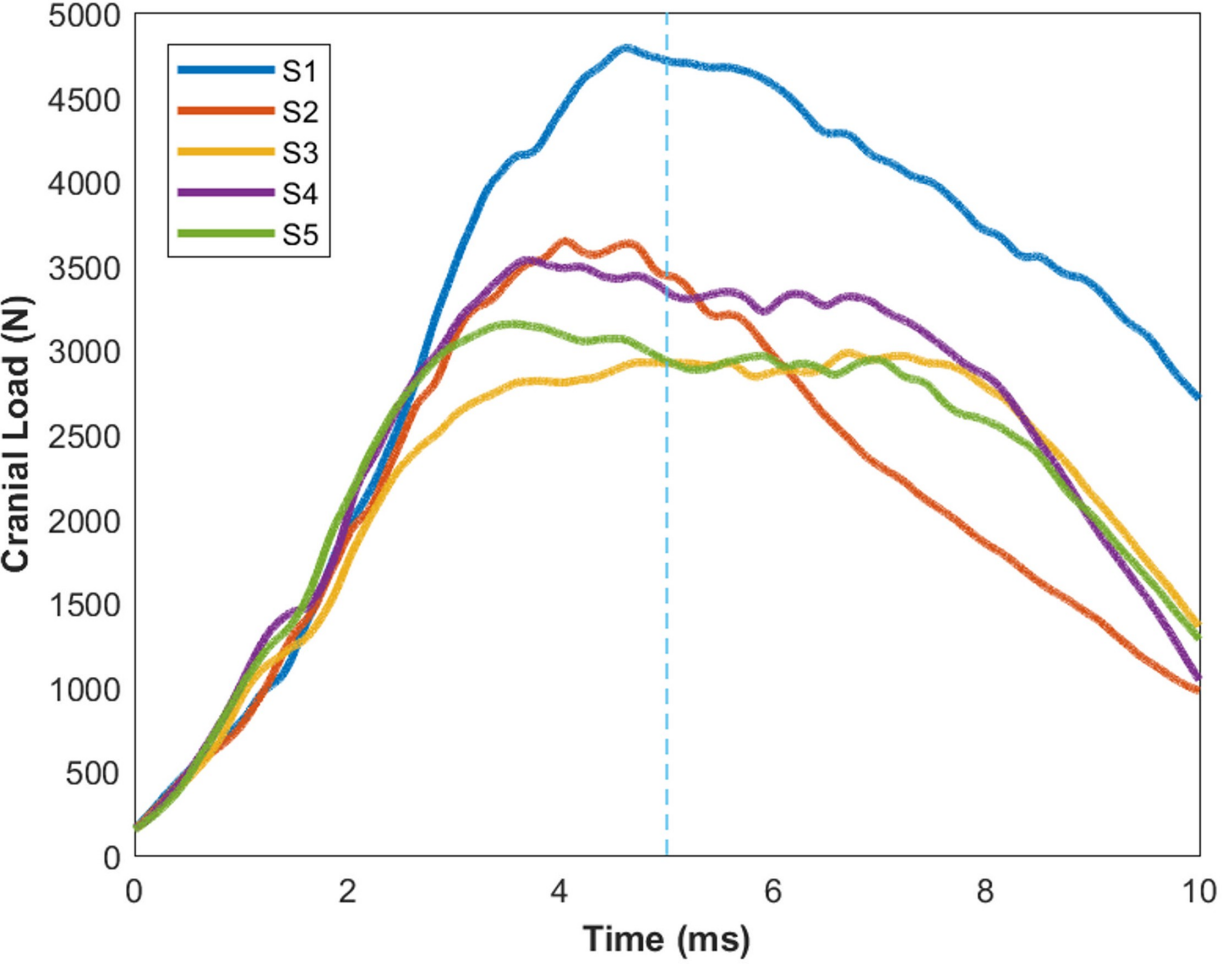
Axial loads measured at the cranial load cell during the experiments. The initial 5 ms (segmented vertical line) of the load traces were used to drive the forward dynamics simulations by applying them to the centre of mass of the C2 segments of the models.

Peak cranial loads measured experimentally ranged from 3.0 to 4.8 kN (Fig 4) with maximal axial displacements of 1.2 to 7.5 mm. One of the six tested specimens (S6) suffered vertebral body fractures at the C2, C3 and C4 levels and was not included in the report of the final parameter values. The genetic algorithm evaluated 100 sample populations over 15 generations of the parameter space with an approximate run time of 10 hours (real-time) per model on a 3.00 GHz v6 Xeon processor with 32 GB RAM.

**Fig 5:**
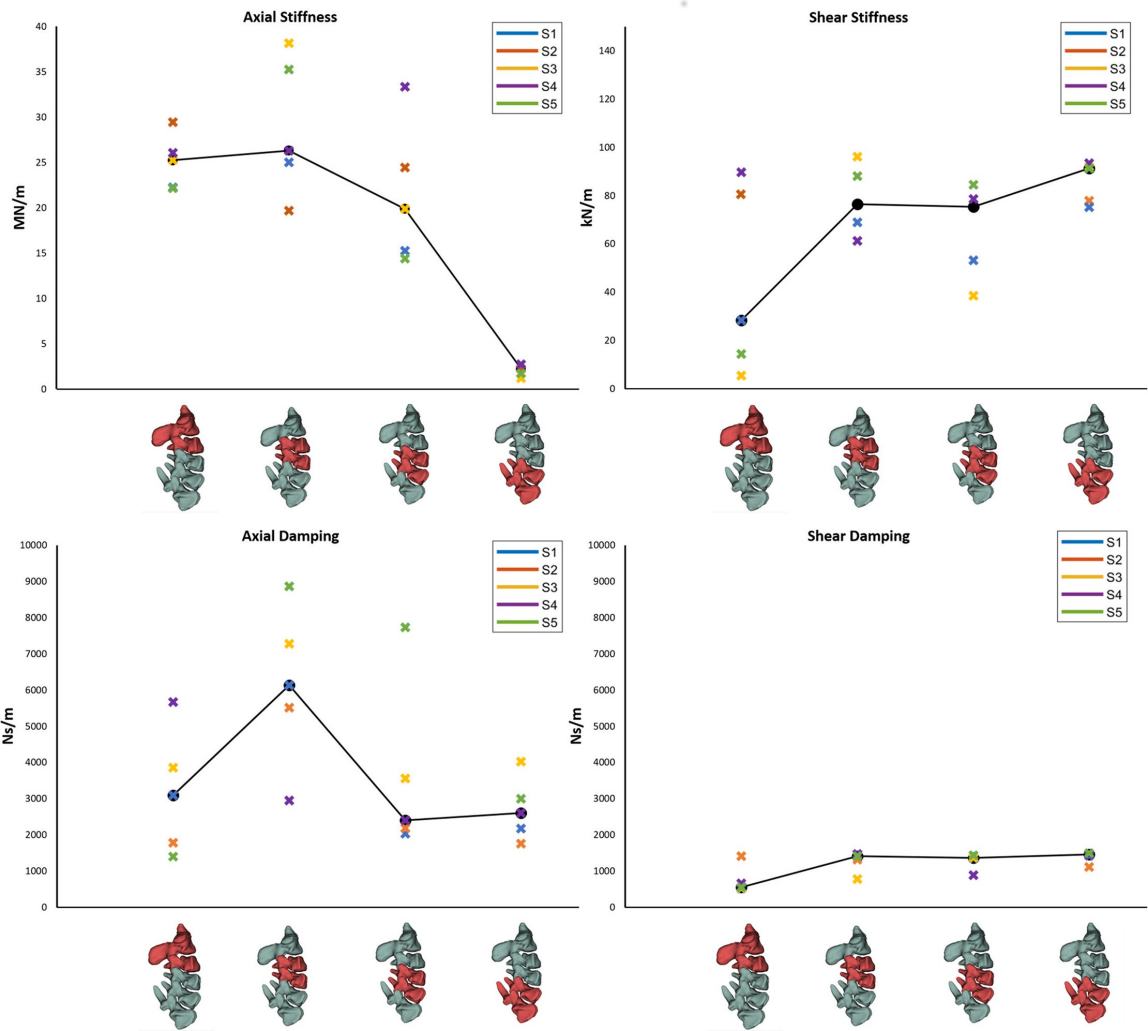
Parameter values identified by the optimisation procedure. Axial stiffness (top left), shear stiffness (top right), axial damping (bottom left) and shear damping (bottom right). Values are shown for each of the cervical spine joints identified by the two red coloured vertebrae on the horizontal axis and for each of the five specimens identified by the legends.

Overall, the estimated values for axial stiffness, axial damping shear stiffness and shear damping across the four joints increased with respect to the initialised values taken from the literature, and ranged between 2.2 to 26.6 MN/m, 2.4 to 6.1 Ns/m, 28.4 to 91.2 kN/m and 0.6 to 1.5 Ns/m respectively (Fig 5, Tables 2 and 3). The average RMSE of the five models was 0.46 mm across the 5 ms between the simulation and measured kinematics (Table 4). The five-fold cross validation showed that interchanging bushing parameter values between models closely replicated model kinematics as tracking errors increasing by 2.5 to 6.4% for specimens S1, S3, S4 and S5, whilst specimen S2 showed a 35.4% increase compared to optimised tracking errors (Table 4).

**Table 2:** Axial stiffness (k) and damping (b) parameter values identified for each specimen specific model of the spinal specimens (S1-S5).

	C2-C3		C3-C4		C4-C5		C5-C6	
Axial	Stiffness (k) N/m	Damping (b) Ns/m	Stiffness (k) N/m	Damping (b) Ns/m	Stiffness (k) N/m	Damping (b) Ns/m	Stiffness (k) N/m	Damping (b) Ns/m
**Initialised values**	1.1×10^6^	10^3^	1.1 10^6^	10^3^	1.1×10^6^	10^3^	1.1×10^6^	10^3^
**S1**	22.2×10^6^	3.1×10^3^	25.0×10^6^	6.1×10^3^	15.2×10^6^	2.0×10^3^	2.7×10^6^	2.2×10^3^
**S2**	29.4×10^6^	1.8×10^3^	19.7×10^6^	5.5×10^3^	24.4×10^6^	2.2×10^3^	2.2×10^6^	1.8×10^3^
**S3**	25.2×10^6^	3.9×10^3^	38.2×10^6^	7.3×10^3^	19.9×10^6^	3.6×10^3^	1.2×10^6^	4.0×10^3^
**S4**	26.0×10^6^	5.7×10^3^	26.3×10^6^	3.0×10^3^	33.3×10^6^	2.4×10^3^	2.7×10^6^	2.6×10^3^
**S5**	22.2×10^6^	1.4×10^3^	35.2×10^6^	8.9×10^3^	14.4×10^6^	7.7×10^3^	1.7×10^6^	3.0×10^3^
**Median**	25.2×10^6^	3.1×10^3^	26.3×10^6^	6.1×10^3^	19.9×10^6^	2.4×10^3^	2.2×10^6^	2.6×10^3^

Initialised values used at the start of the optimisation are presented in the first row.

**Table 3:** Shear stiffness (k) and damping (b) parameter values identified for each specimen specific model of the spinal specimens (S1-S5).

	C2-C3		C3-C4		C4-C5		C5-C6	
Shear	Stiffness (k) N/m	Damping (b) Ns/m	Stiffness (k) N/m	Damping (b) Ns/m	Stiffness (k) N/m	Damping (b) Ns/m	Stiffness (k) N/m	Damping (b) Ns/m
**Initialised values**	63.0×10^3^	10^3^	63.0×10^3^	10^3^	63.0×10^3^	10^3^	63.0 10^3^	10^3^
**S1**	28.4×10^3^	0.5×10^3^	69.0×10^3^	1.5×10^3^	53.3×10^3^	1.4 10^3^	75.3 10^3^	1.4 10^3^
**S2**	80.6×10^3^	1.3×10^3^	76.4×10^3^	1.3×10^3^	75.5×10^3^	1.4 10^3^	77.8 10^3^	1.1 10^3^
**S3**	5.5×10^3^	0.5×10^3^	96.1×10^3^	0.8×10^3^	38.5×10^3^	1.4 10^3^	92.0 10^3^	1.5 10^3^
**S4**	89.7×10^3^	0.7×10^3^	61.3×10^3^	1.5×10^3^	78.6×10^3^	0.9 10^3^	93.5 10^3^	1.5 10^3^
**S5**	14.5×10^3^	0.6×10^3^	88.0×10^3^	1.4×10^3^	84.5×10^3^	1.4 10^3^	91.2 10^3^	1.5 10^3^
**Median**	28.4×10^3^	0.5×10^3^	76.4×10^3^	1.4×10^3^	75.5×10^3^	1.4 10^3^	91.2 10^3^	1.5 10^3^

Initialised values used at the start of the optimisation are presented in the first row.

**Table 4:** Root mean square errors (RMSEopt–Column 2) across the 15 tracking markers between measure and simulated kinematics during the optimisation procedure.

Specimen number	RMSE_opt_ (mm)	RMSE_val_ (mm)	RMSE_lit_ (mm)	Calibration error (mm)
**S1**	0.46	0.47	2.59	0.24
**S2**	0.33	0.45	2.28	0.12
**S3**	0.58	0.59	2.08	0.17
**S4**	0.51	0.53	2.60	0.50
**S5**	0.44	0.46	2.30	0.29

Errors are also presented for the five-fold cross validation (RMSEval−Column 3) and model evaluations using joint viscoelastic values from the literature (de Bruijn et al., [9]) that were used to initialise the models at the start of each optimisation (RMSElit−Column 4). Finally, the calibration error of the motion capture system for each experimental measurement is presented for comparison (Column 5).

**Fig 6:**
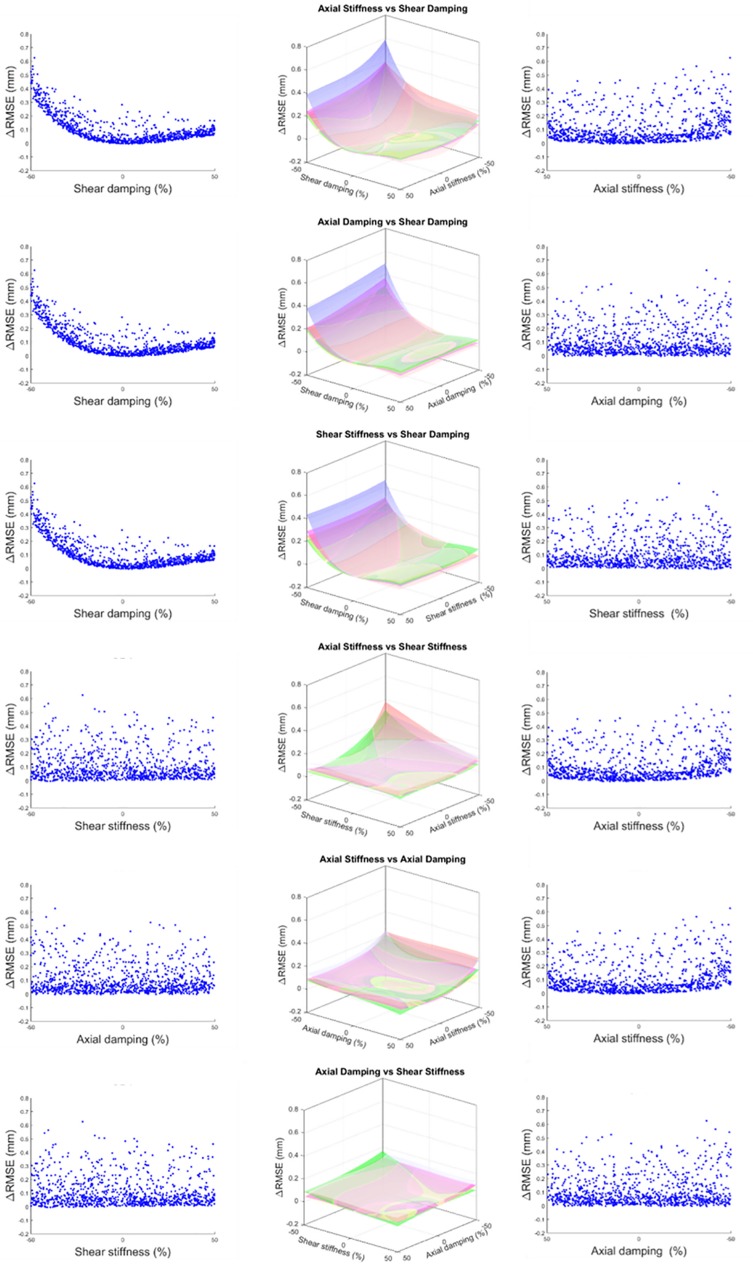
Results of the 1000 sample Monte Carlo sensitivity analysis for the five specimen-specific models. Results are presented in order of their effect on the ΔRMSE (largest to smallest). The axonometric view (central column) shows the response of the five models as the interpolated 3^rd^ degree polynomial surfaces between the six possible parameter combinations. Left and right columns show the projection of each axis of the parameter variation against the ΔRMSE on their respective sides for S1 as an example of the response.

The models showed a similar response to individual and combined parameter variations during the sensitivity analysis (Fig 6). Changing shear damping and axial stiffness parameters in isolation resulted in the largest increases of RMSE by 0.2 to 0.4 mm. When shear damping and axial stiffness were concurrently perturbed models showed the largest combined effect on RMSE ranging between 0.4 and 0.6 mm (Fig 6: 1^st^ and 2^nd^ rows). Perturbations in anteroposterior joint locations resulted in RMSE increases <0.1 mm.

**Fig 7:**
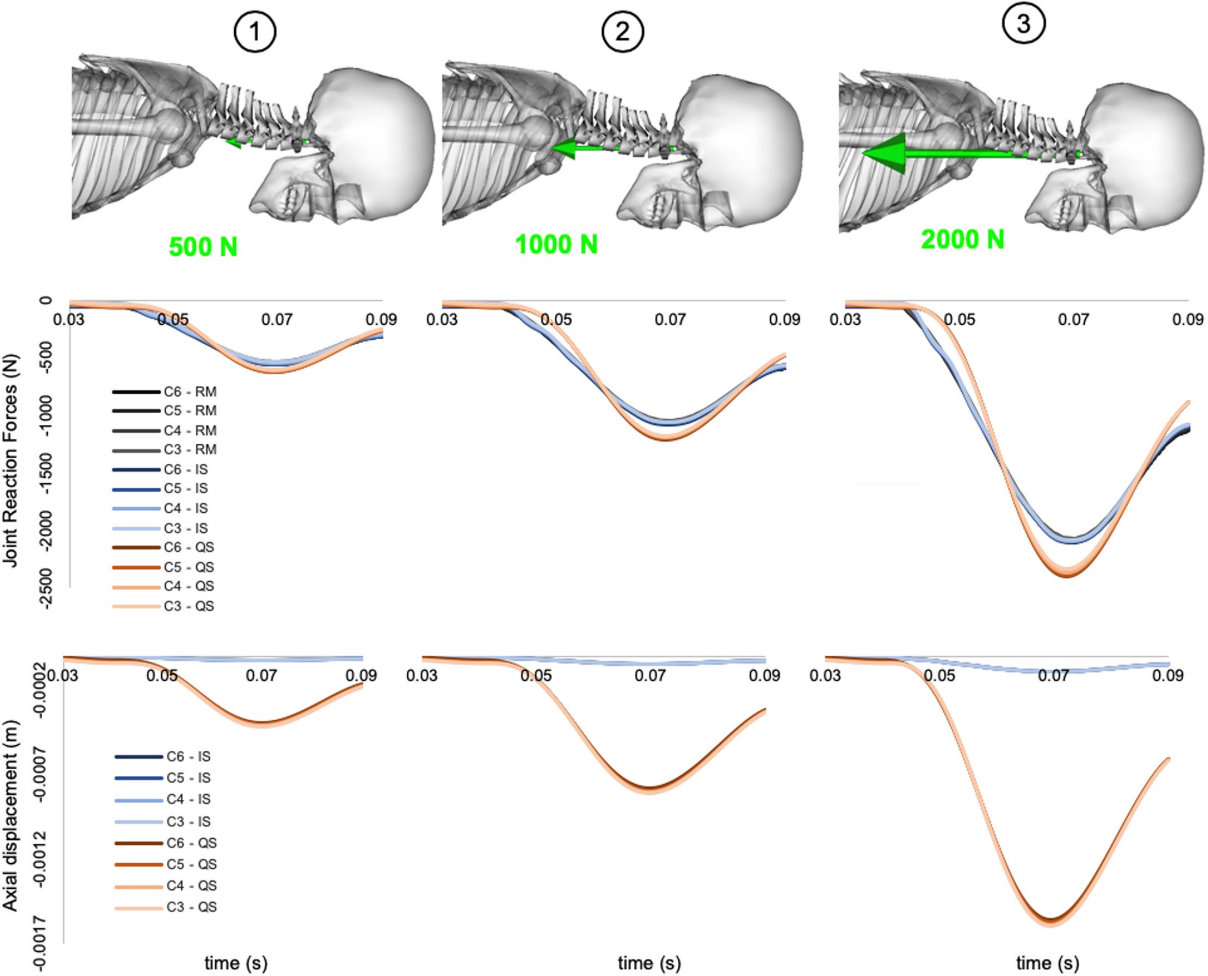
Forward dynamic results of theoretical injurious sporting scenario. Comparison of internal joint loads (Row 2) and resulting joint displacements (Row 3) calculated from the three versions of the musculoskeletal model and across three loading conditions (Row 1). Only joint loads are displayed for the Rugby Model (RM) as the kinematic constraints do not allow for joint translation which is displayed for Impact Specific (IS) and Quasi-Static (QS) versions of the model.

The model comparison showed a similar response during the sub-injurious scenarios, whilst the injurious scenario highlighted a different behaviour. The impact specific model and the original “Rugby Model” yielded similar peak joint loads, whilst the quasi-static model estimated 13 to 15% higher compressive loads for the three tested impact conditions (Fig 7: 2^nd^ row). The resulting joint compressions of the bushing model with the new parameters allowed smaller displacements (<0.1 mm) compared to the model implemented with the previous parameters (0.4 to 1.5 mm) (Fig 7: 3^rd^ row).

## Discussion

The purpose of this study was to identify and validate cervical spine viscoelastic joint parameters under impulsive axial impact conditions, and integrate them in a musculoskeletal model for the analysis of injury mechanisms. Specimen specific musculoskeletal models of porcine cervical spines were used as surrogates to human specimens to estimate joints’ axial and shear viscoelastic values. Combined *in vitro* and *in silico* approaches allowed to identify the parameters that describe the viscoelastic response of individual cervical joints, and successfully apply them for the analysis and simulation of a sporting scenarios.

### Viscoelastic parameter estimation

The estimated stiffness values for axial compression increased by one order of magnitude from the initialisation values for all joints except the most caudal (C5-C6). Compressive damping as well as shear stiffness and damping also showed an increasing trend but remained within the same order of magnitude as the initialising values [9]. The large increase of axial stiffness values is likely to be related to the high impulsive load applied to the spine. The viscoelastic behaviour of the intervertebral disc has been characterised by its non-linear response to loading especially in axial compression which is a degree of freedom highly affected by the poroelastic properties of the disc [21, 22, 31, 46]. *In vitro* studies have measured increased apparent stiffness of intervertebral discs and functional units when subjected to higher loading rates indicating grater energy storage than energy dissipation under these conditions [21, 22].

The musculoskeletal models of the presented study used a parallel arrangement of linear stiffness and damping elements (Kelvin-Voight model) to approximate the dynamics of the spine. This representation of the intricate dynamic behaviour of the intervertebral disc has been utilised previously [9, 10] and its ease of implementation into musculoskeletal models provides an added benefit for their use. More complex viscoelastic models of the intervertebral disc may be used in musculoskeletal models, however the inclusion of additional parameters over multiple levels of the spinal structure could lead to overfitting or generate multiple solutions that make the interpretation of the results difficult. There is potential that in future studies more detailed viscoelastic models can be explored however this initial use of a simple Kelvin-Voight model resulted in good estimation of the general intervertebral disc properties under dynamic loads.

Linear bushings, however, only approximate regions of the inherent non-linear behaviour of the intervertebral disc’s force-displacement curves. Thus, the higher estimated values represent a steeper portion of the force-displacement curve of the intervertebral discs caused by the high loading rate of sporting collisions. Median compressive stiffness values of the C2-C3 to C4-C5 joints were within similar values of 19.9 to 26.3 MN/m compared to the stiffness for the C5-C6 joint of 2.2 MN/m. The lower axial stiffness values found at the most caudal joint (C5-C6) are attributed to the experimental and computational constraints during the experiments as well as the relative position of the joint with respect to the axial force vector applied at the C2 vertebra of the specimen. Full kinematic constraints on the C6 body of the models may have neglected small motions experienced in the experiment, and thus underestimated the joint stiffness. Additionally, the caudal section of the cervical spine displays greater lordosis which cause a rotation of the joint reference system in the sagittal plane (Fig 2) and directs the vector of the axial force at a more of a shear angle to the C5-C6 joint. The effect of such anatomical change would be to transfer the load in a more anterior direction with respect to the joint. Similarly, optimal values in the shear direction were within closer ranges for the C3-C4 to C5-C6 joints compared to the C2-C3 joint. The constraints of the most cranial (C2) and caudal (C6) segments of the specimens allowed the intermediate vertebrae to be loaded in a more physiologic manner as they were experimentally unconstrained. This resulted in two joint levels C3-C4 and C4-C5 to be displaced with no experimental constraints acting on any of their segments. Therefore, due to the large sagittal angle of the C5-C6 joint to the axial force vector, it is suggested that the median values of the C3-C4 and C4-C5 joints’ axial and shear viscoelastic parameters estimated in this study, should be used across cervical spine joints in multibody models investigating impulsive axial impacts to the head. This strategy was adopted in the analysis of joint loads by implementing the stiffness and damping values in bushing parameters at the cervical spine joints of the validated “Rugby Model” [44].

Investigations on the dynamic stiffness of individual joint levels of multi-jointed cervical spines have been limited. The increased compressive stiffness estimated for these specimens under the large impulsive loads logically follow results from static and quasi-static experiments on single joint units [29, 31, 47]. The studies by Panjabi et al. [47] and Moroney et al. [29] used incremental static loads up to a physiological loading range of 50 N to study the stiffness of cervical motion segments. Stiffness values from the two studies differed substantially with 141 vs 1318 kN/m and 34 vs 131 kN/m for axial compression and anteroposterior shear respectively. The static results of Moroney et al. [29], however, closely matched the quasi-static stiffnesses by Shea et al. [31] obtained from loads up to 2000 N. Both of these studies reported large variability in stiffness between specimens. This suggests that the range of viscoelastic parameter values found in this study could be caused primarily by the physiological inter- and intra-specimen variability rather than the optimisation search. Musculoskeletal models of the human neck used in automotive research [9, 48, 49] have used the compressive stiffness values of these experimental studies when investigating injuries during collisions. However, the applicability of these values from static and quasi-static experiments to analyses of dynamic events remains an open question. Damping values of 1000 Ns/m were selected to sufficiently attenuate head acceleration, however it was believed these values may still be too low [48], which supports the larger damping values estimated in this study.

The experimental set-up of this study applied a higher compressive preload (152 N) to the physical specimens compared to previous experiments of 10 N and 42 N [29, 47]. A larger preload, more representative of that experienced in-vivo, does stiffen the cervical spine specimen prior to impact compared to specimens impacted without a preload/follower-load [33]. The higher preload [33, 35] together with the impulsive loading would support the significantly higher compressive stiffness increase compared to damping of the intervertebral discs that was estimated. This was supported by the sensitivity analysis where lower axial stiffness values resulted in higher tracking errors. Investigations of intervertebral disc mechanical response over increased loading rates have demonstrated that energy dissipation decreases at higher rates compared to energy storage caused by the fluid-solid phase of the disc [21, 22, 46]. However, the fluid-solid phase of the disc as a function of disc deformation is difficult to examine due to its complex tissue matrix structure, internal and peripheral fluid flow and endplate diffusion. The significantly increased compressive stiffness over shear stiffness is supported because the axial compression degree of freedom is a disc deformation mode where fluid flow effects are greater than in shear [22].

An acceptable parameter fit tested by the five-fold cross validation displayed significantly closer tracking results (RMSE_val_) compared to when the models were evaluated using parameter values from the literature (RMSE_lit_) [9] (Table 4). The smaller increase of RMSE_val_ compared to RMSE_lit_ from RMSE_opt_ supports previous findings that during impulsive axial loading the cervical spine responds in a stiffer manner. The Monte Carlo analysis also showed that models’ responses were sensitive to decreases in axial stiffness (*k*_*y*_). Perturbations in shear damping (*b*_*x*_) combined with axial stiffness (Fig 6: Row 1) resulted in the largest relative increases in tracking errors (ΔRMSE). Lower shear damping appears to have a large effect on the models’ performance (Fig 6: Row 1). During these impulsive axial impacts the cervical spines showed a rapid but non-injurious anterior buckling response as previously observed by Nightingale et al. [50]. The anterior shear motion of the vertebrae caused by the buckling of the specimens, however, did not lead to injuries because the applied load was chosen to be sub-catastrophic. This also indicates that the energy transmitted from the axial impact causing the anterior vertebral motion was dissipated quickly. These results highlight the importance of anterior-posterior joint damping parameters used in musculoskeletal models analysing cervical spine injury mechanisms of axial impacts. In fact, the inclusion of lower values of shear damping in the models may result in an excessive anterior motion of the vertebrae, and in a subsequent erroneous prediction of the injurious events (i.e. joint dislocation).

### Models comparison and application to injury prevention analyses

A reliable estimation of joint loads and resulting joint kinematics during impacts is key for the analysis of the injury mechanisms and estimation of injury risk. This becomes extremely important in sporting scenarios where real-world interventions, which aim to minimise injury occurrence, are informed by the output of injury mechanisms analyses. There is therefore a pressing need to use accessible computational tools, such as musculoskeletal models, capable of estimating internal joint loading and simulating injurious scenarios without adding excessive complexity. In fact, it is very challenging to directly integrate conventional *in vivo* measurements of sporting activities with finite element analyses. Currently more detailed finite element analyses are often driven by *in vitro* experimental loads and kinematics that do not adequately describe the *in vivo* behaviour during these impact events. Therefore musculoskeletal modelling is a valuable link between real-world measurements and more complex structural analyses and provides appropriate boundary conditions for finite element analyses [18].

The viscoelastic parameters estimated in this study, and their integration in a previously validated musculoskeletal model (i.e. the “Rugby Model”), provide a valid and accessible tool for such analyses. In fact, the comparison with previous models clearly shows the importance of using impact-specific bushing parameters to estimate realistic joint loads and simulate injurious events. The three versions of the “Rugby Model” tested under axial impacts revealed differences in their simulated kinematics but comparable loading patterns. Similar peaks of compressive load between the impact-specific “Rugby Model” and the original “Rugby Model” are expected. This is due to the high axial stiffness value mimicking the response of the translationally constrained joints of the “Rugby Model”. The higher peak loads showed by the non-impact-specific “Rugby Model” could be attributed to a larger effect of the damping component and lower stiffness values. This illustrates the benefit of using impact-specific parameters compared to bushings validated in quasi-static conditions when used in impulsive events. From a joint displacement perspective, the model using quasi-static bushing parameters showed joint displacements which were near failure values of 0.84 mm [51]. As a result, the use of bushing parameters not validated for the analysis of impact events can misrepresent the resulting joint kinematics due to lower stiffness values, and therefore indicating erroneous injury mechanisms.

### Experimental and modelling assumptions

The experimental and modelling assumptions of this study must be highlighted. Firstly, the simulations were driven only by a compressive axial load applied at the C2 vertebrae as the experimental load cell was uniaxial and the applied load was delivered primarily in the axial direction via the impactor. This may have neglected anteroposterior or medio-lateral shear loads that were not measured by the load cell. Cyclic preconditioning, such as series of lower magnitude axial loads, was not performed in case of specimen damage. Such preconditioning would affect the fluid content of the intervertebral discs and possibly influence the response of the spine under axial load. Preconditioning is commonly done under similar loads to the ones used for testing, however in the presented experiment a series of lower magnitude axial loads would have the potential to weaken the specimens prior to testing. Another source of error was potentially introduced by the natural resonant frequency of the tracking clusters due to their lever arms. The virtual markers were positioned at constant distances from the geometry of the models however, the experimental clusters may have experienced lag between the vertebral movement and the tracking cluster displacement during impact. The genetic algorithm minimised the tracking errors between the measured and simulated marker kinematics by optimising the 16 axial and shear joint stiffness and damping parameters of the models (Table 4). The similarity of this problem with automotive suspension design problems [52, 53] and the genetic algorithm’s ability to search the parameter space for solutions was the reason the algorithm was chosen. Finally, porcine specimens have been evaluated as surrogates to human specimens in injury mechanism studies [54–56]. Furthermore, they provide a more homogeneous sample allowing for a better controlled experimental design without the effect of confounding factors such as age and level of degradation [46]. This is important for injury mechanism analysis as these factors can influence the effects of rapidly applied loads experienced by a young sporting population. However, the use of porcine specimens for the investigation may not be entirely representative of the functional joint behaviour of human specimens.

## Conclusion

This is the first study providing cervical spine joints (C2-C6) viscoelastic parameters for the analysis of injury mechanisms during axial impacts. The bushing (Kelvin-Voight) parameters were estimated via combined *in vitro* experimental and *in silico* musculoskeletal modelling approaches. Specimen-specific cervical spine models were created and validated against *in vitro* 3D kinematic data of high impact loading situations. Results showed higher values of axial stiffness in unconstrained joints compared to previous values found in the literature derived from static and quasi-static experiments. Researchers should also be aware of the sensitivity of spinal models to low values of axial stiffness and shear damping when investigating axial impacts to the spine. Finally, this study provides the first proof-of-concept that a musculoskeletal modelling approach can be used to analyse cervical spine injury mechanisms by allowing the estimation of internal joint loads and simulating realistic joint kinematics during in sporting scenarios.
